# Biomechanical Assessments in Woodwind Musicians: A Systematic Review

**DOI:** 10.3390/healthcare11111621

**Published:** 2023-06-01

**Authors:** Javier López-Pineda, María Carmen Rodríguez-Martínez, Rosa Gómez-Rodríguez, Lucía García-Casares, Natalia García-Casares

**Affiliations:** 1 Clínica López & Cruzado, 29720 La Cala del Moral, Spain; info@clinicalopezycruzado.com; 2 Instituto de Posturología y Promoción de la Salud del Músico (IPPSM), 29720 La Cala del Moral, Spain; 3 Department of Physiotherapy, Faculty of Health Sciences, University of Malaga, 29071 Malaga, Spain; 4 Instituto de Investigación Biomédica de Málaga (IBIMA), 29590 Malaga, Spain; nagcasares@uma.es; 5 Hospital Universitario Fundación Jiménez Díaz, 28040 Madrid, Spain; rosa.grodriguez@quironsalud.es; 6 Conservatorio Elemental de Música Santa Cecilia, 11130 Chiclana de la Frontera, Spain; lugcasares@gmail.com; 7 Departament of Medicine, Faculty of Medicine, University of Malaga, 29010 Malaga, Spain; 8 Centro de Investigaciones Médico-Sanitarias (CIMES), 29071 Malaga, Spain

**Keywords:** biomechanical assessments, musculoskeletal disorders, pressure sensors, surface electromyography, musicians, woodwind instruments

## Abstract

Biomechanical methods are frequently used to provide information about the kinematics and kinetics of posture and movement during musical performance. The aim of this review was to identify and analyze the biomechanical methods performed on woodwind musicians to understand their musculoskeletal demands. A systemic review was carried out following the guidelines of the document Preferred Reporting Items for Systematic Reviews and Meta-Analyses (PRISMA). It was registered in PROSPERO (code 430304).The databases PubMed, Cochrane, CINAHL, Scopus, and Web of Science were consulted between January 2000 and March 2022. The search in the databases identified 1625 articles, and 16 different studies were finally included in the review, with a sample size of 390 participants. Pressure sensors, surface electromyography, infrared thermography, goniometry in two dimensions, and ultrasound topometry in three dimensions were biomechanical methods useful to broaden the knowledge of musculoskeletal demands during musical practice. Piezoresistive pressure sensors were the most widely used method. The great heterogeneity of the studies limited the comparability of the results. The findings raised the need to increase both the quantity and the quality of studies in future research.

## 1. Introduction

Performing arts biomechanics emerged as a specialty within performing arts medicine, and it is responsible for quantifying the musculoskeletal demands of artistic tasks [1].In the area of music, instrumented biomechanical methods offer precise information on kinematics and kinetics, allowing greater insight into the movement patterns of musicians during performance [2]. This is of special relevance, considering that playing posture, repetitive movements, long study sessions, as well as the musician’s own technique are risk factors for the development of musculoskeletal disorders (MSDs) related to musical practice [3,4].

Therefore, the first step for both treatment and prevention is to understand the underlying reasons and associated risk factors [5]. In this regard, the data obtained through biomechanical methods offer precise information to understand and minimize the risk of injuries [6].MSDs usually manifest as muscle overexertion, afflicted tendons, muscle tension, and fatigue [4]. In fact, musculoskeletal symptoms can range from discomfort to severe or permanent conditions that can affect the performance quality and even prevent the musician from playing [7,8,9].

Of the reported point prevalence of musculoskeletal complaints related to playing, 12-month prevalence ranges between 41 and 93%, whereas lifetime prevalence ranges between 62 and 93% [10]. The scientific literature includes a variety of MSDs related to musical practice, such as back pain and neck pain, shoulder tendinopathy (for example, rotator cuff tendinopathy), epicondylitis and epitrocleitis, De Quervain’s tenosynovitis, digital stenosing tenosynovitis, ulnar nerve entrapment at the elbow, and movement disorders such as focal dystonia (in the hand and the orofacial musculature) [9,11,12].With regard to the embouchure in wind musicians, different studies have described various pathologies (or disorders) such as fatigue or tear of the orbicularis oris muscle, pain in the temporomandibular joint, lip tremor, and focal dystonia [9,13,14,15].

Several reviews have identified biomechanical evaluations on musicians [1,5,6]. Kelleher et al. [1] identified and categorized the biomechanical assessments in violinists, violists, cellists, and double bassists. The researchers found that the most widely used methods were surface electromyography (SEMG) and kinematic studies, which were mostly based on the analysis of movement in three dimensions (3D) using three cameras and reflective markers. This technique, known as photogrammetry, is a useful tool for studying movement patterns, joint angles, and marker velocities [1]. Schemmann et al. [5] identified evaluations based on quantitative studies of violinists and violists. Most of the kinematic studies were based on photogrammetry, and electromyography (EMG) was one of the most commonly used methods, being applied in the upper extremity, the neck, and the jaw [5]. Finally, Herrmann et al. [6] identified quantitative studies on musculoskeletal demands in brass musicians. The most widely used methods were kinematic assessments in two and three dimensions, SEMG, and kinetic evaluations [6]. It is worth noting that no study conducted a review of biomechanical assessments in woodwind musicians.

EMG allows to determine the amplitude and moment of the muscle activation while the interpreter plays the instrument [1], resulting in special interest in the various biomechanical methods applied to the musician. Kjelland [16] carried out a review of the application of SEMG and EMG biofeedback. More recently, Overton et al. [17] performed a systematic review of the available evidence of the muscle activity of the neck, spine, and shoulder musculature using EMG in instrumentalists [17]. SEMG and EMG biofeedback are effective tools as methods for the diagnosis and improvement of interpretative skills [16].

On the other hand, infrared thermography (IT) represents a non-invasive, rapid, and portable technique that measures the temperature of the skin with no risk of radiation. In recent years, there has been greater use of thermal cameras for the diagnosis of various pathologies such as neuropathies, peripheral vascular disease [13,18], inflammatory diseases, complex regional pain syndrome, and rheumatic diseases such as osteoarthritis, rheumatoid arthritis and fibromyalgia [19,20]. IT has also been used in sports to detect thermal asymmetries in various regions of the body that can help in the early detection of musculoskeletal overloads and fatigue, as well as in injury prevention [21]. In the area of music, IT has already been shown to be a useful tool in the diagnosis of pathologies that affect the masticatory muscles and the orofacial musculoskeletal structures [18,22]. However, most of the studies were conducted on violinists and violists, and very few studies have investigated the relationship between playing a wind instrument and temporomandibular disorders (TMDs), even though playing a wind instrument requires the participation of more orofacial muscles than playing a string instrument [18].

Musicians are an elite occupational group comparable to professional athletes and represent a significant proportion of the performing arts sector. However, their occupational health is often not considered, and research considering this group is limited [17]. It is for this reason that a better understanding of the biomechanics related to musical performance may have important implications in the treatment and prevention of injuries or MSDs related to musical practice and performance.

According to these considerations, this systematic review aims to identify and analyze the biomechanical methods performed on woodwind musicians to understand their musculoskeletal demands.

## 2. Materials and Methods

### 2.1. Search Strategies

The PubMed, Cochrane, CINAHL, Scopus, and Web of Science databases were consulted. The database searches identified 1625 studies published from January 2000 to March 2022 using the following search strategy: (motion OR movement OR posture OR electromyography OR infrared thermography OR pressure sensors OR piezoresistive sensors OR force sensors OR three dimensional) AND (wind instrumentalists OR woodwind players OR clarinet OR saxophone OR flute OR bassoon OR oboe). A systemic review was carried out following the guidelines of the document Preferred Reporting Items for Systematic Reviews and Meta-Analyses (PRISMA) [23]. It was registered in PROSPERO (code 430304).

### 2.2. Elegibility Criteria

Inclusion criteria were: studies that included woodwind players (clarinetists, saxophonists, flutists, bassoonists, and oboists) and that used biomechanical methods to describe kinematic, kinetic, or physiological aspects related to musicians’ posture or movement. Single case articles were included since they comprised biomechanical methods used to better understand the musculoskeletal demands during musical practice, thus responding to the objective of this systematic review.

Exclusion criteria were: reviews, letters to the editor, conference abstracts, and articles published in languages other than English. Articles that did not meet the objective of the review (use of other non-biomechanical methods, analysis of movement patterns and musical expressiveness or temporal precision of the musical performance, study of respiratory parameters and anxiety) were also excluded.

### 2.3. Study Selection

Two reviewers independently reviewed studies for their potential inclusion against the eligibility criteria. Any disagreement was resolved by arbitration of a third reviewer.

### 2.4. Data Extraction

Two reviewers retrieved the data independently. Data extraction was carried out using a single form with the following information: first author and year of publication, characteristics of the participants (number of subjects, age, gender, state of health, type or group of musical instrument, occupation (professional, student, amateur) and years of practice), biomechanical methods, objectives, musical activity, other evaluations (non-biomechanical), results, and conclusions.

### 2.5. Quality Assessment of Included Studies

The studies included were assessed for quality using the checklist Strengthening the Reporting of Observational Studies in Epidemiology (STROBE) [24]. The STROBE statement is a checklist of 22 items considered essential for the proper communication of observational studies [24].

## 3. Results

### 3.1. Selected Studies

**Figure 1:**
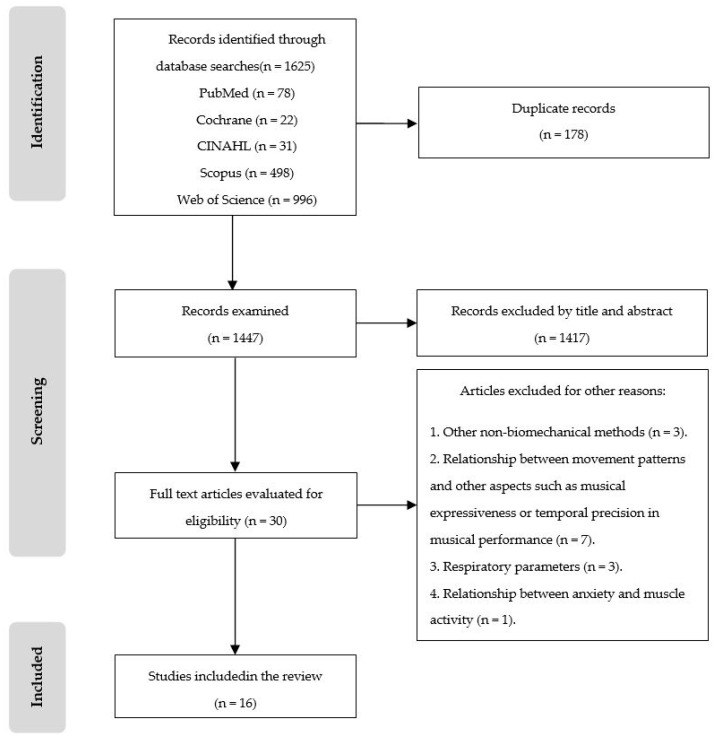
PRISMA flowchart of studies selection.

After the removal of duplicate records, a total of 1447 were reviewed screening titles and abstracts. Of these, 1417 articles were excluded because they did not deal with the subject studied. For example, studies on airflow measurement, reed vibration, geography, and protein were excluded. Finally, 30 were deemed to warrant full-text evaluations. After analysis of the remaining 30 studies, 16 articles met the inclusion criteria and were included in this review (Figure 1).

### 3.2. Characteristics of the Participants of the Included Studies

The studies included a total of 390 subjects aged between 15 and 60 years. The characteristics of the participants are shown in Table 1. Four of the studies did not specify gender [25,26,27,28], and the other four did not specify age [26,28,29,30]. The participants were professional, student, or amateur musicians, although this variable was not specified in five studies [27,30,31,32,33]. The clarinet was the most frequently used instrument, appearing in twelve studies [25,26,27,28,31,32,33,34,35,36,37,38].

### 3.3. Summary of Selected Studies

Table 2 specifies the following information for each study: authors, biomechanical methods, objectives, musical activity, results, other non-biomechanical evaluations, conclusions, and scores obtained by applying the STROBE tool to assess its quality.

**Table 1:** Participants characteristics.

Authors (Year)	N	Age	Gender	Estate of Health	Type/Group of Instrument	Professional, Student, Amateur	Years of Practice
Ackermann et al. (2014) [34]	113	34.1 *	68M/45F	N/A	Clarinet (12), bass clarinet (2), oboe (11), English horn (3), saxophone (3), flute (23), recorder (1), piccolo (2), shakuhachi (1), bassoon (11), contrabassoon (3), french horn (10), trombone (9), bass trombone (2), trumpet (16), and tuba (4)	Professionals and students	N/A
Baadjou et al. (2017) [35]	20	18–60	9M/11F	Healthy	Clarinet	Professionals and students	19.4 *
Barros et al. (2018) [25]	30	18–49 25.5 *	N/A	N/A	Clarinet	Professionals and students	8–37
Clemente et al. (2018) [29]	1	N/A	F	Periapical lesion (tooth 21)	Saxophone	Professional	N/A
Clemente et al. (2018) [30]	1	N/A	M	TMD	English horn and oboe	N/A	N/A
Clemente et al. (2018) [31]	1	30	F	TMD	Clarinet	N/A	N/A
Clemente et al. (2019) [26]	28	N/A	N/A	No pain	Clarinet (7), oboe (2), saxophone (7), bassoon (4), trumpet (6), french horn (1), trombone (1). Transverse flute (1) and bisel flute (1)	Students	N/A
Clemente et al. (2019) [27]	3	>18	N/A	Malocclusion	Clarinet (1), tuba (1), and bassoon (1)	N/A	>10
Clemente et al. (2019) [28]	10	N/A	N/A	No pain	Clarinet (5) and saxophone (5)	Students	N/A
Clemente et al. (2020) [39]	77	18–31	41M/36F	N/A	Woodwind (27), brass (22), and strings (28)	Students	>10
Franz et al. (2020) [36]	8	20.0 * (students)– 33.0 * (professionals)	3M/5F	Healthy	Clarinet	Professionals and students	N/A
Gotouda et al. (2007) [32]	33	15–27	12M/21F	Pain in TMJ and jaw muscles.	Clarinet (7), oboe (3), saxophone (2), bassoon (2), trumpet (8), french horn (4), trombone (4), tuba (2), and euphonium (1)	N/A	N/A
Hofmann y Goebl (2016) [37]	23	19–45 27 *	13M/10F	N/A	Clarinet	Professionals and students	N/A
Piatek et al. (2018) [40]	14	18–38 25.86 *	7M/7F	No MSD	Saxophone	Students and amateurs	<5 to >8
Smyth y Mirka (2021) [33]	8	18–30	1M/7F	Healthy	Clarinet	N/A	>5
Young y Winges (2017) [38]	20	26.2 *	7M/13F	Healthy	Clarinet	Professionals and students	N/A

F, female; M, male; N/A, not available; MSD, musculoskeletal disorder; TMD, temporomandibular disorder; TMJ, temporomandibular joint. * Mean value.

### 3.4. Biomechanical Methods

The use of pressure sensors to measure the force exerted was the most widely used method, being used in eight studies [26,27,28,29,30,31,33,37], followed by the use of SEMG to assess muscle activation, which was used in six of the studies [32,33,34,35,36,38], and by the IT which was used in five of them [25,27,30,31,39]. These biomechanical methods are categorized in Table 3.

### 3.5. Muscles Analyzed by SEMG

Table 4 shows the muscles that were analyzed by SEMG.

## 4. Discussion

The aim of this review was to identify which biomechanical methods were performed on woodwind players to understand their musculoskeletal demands. The findings showed that various assessments were used where pressure or force sensors were the most frequent [26,27,28,29,30,31,33,37], followed by SEMG [32,33,34,35,36,38], IT [25,27,30,31,39], 2D goniometry [35], and 3D ultrasound to pometry (UT) [40]. 

In previous reviews, Herrmann et al. [6] identified quantitative studies involving biomechanical assessments in brass players, while the reviews by Kelleher et al. [1] and Schemmann et al. [5] identified the biomechanical assessments in strings musicians (violinists, violists, cellists, and double bass players). To our knowledge, this is the first systematic review conducted solely on woodwind musicians to identify and summarize findings on biomechanical methods. 

### 4.1. Pressure Sensors

According to the findings of this review, kinetic analysis was based on the measurement of finger force while fingering [37], the force of the right thumb while supporting the weight of the instrument [33], and, more frequently, the force exerted by the musculature that participates in the embouchure [26,27,28,29,30,31]. The clarinet was the most frequently tested instrument, appearing in six of the eight studies that used pressure sensors [26,27,28,31,33,37]. 

There is great heterogeneity in the included studies, which makes it impossible to compare the results. For example, various studies measured the force exerted by the upper incisors in single-reed instruments (clarinet and saxophone) [26,27,29,31], but no uniformity was found regarding the best location for the sensors, which must be superimposed so that they occupy the upper surface of the mouthpiece of the instrument. In this way, the same incisor could be exerting pressure over two different sensors. Moreover, two studies measured the lower lip force during embouchure [26,28], but the number of participants was very small, and none accurately described the musical task. As for the double-reed instruments (oboe, English horn, and bassoon), the researchers measured the pressure exerted by the upper and the lower lip [26,30], but the number of participants for each of the instruments considered was very small.

Therefore, due to the small number of studies, the small number of participants and the scarce methodological information (such as information referring to the characteristics, calibration, and location of the sensors or the musical task performed), as well as the lack of uniformity expressing the measurement units (Newton or grams), the results cannot be compared to reach conclusions. Despite this, pressure sensors have been shown to be useful as a complementary diagnostic tool in four studies [27,29,30,31] according to various pathologies: apical lesion [29], malocclusion [27], and TMD [30,31].

Additionally, only one study measured the force exerted by the fingers during fingering, using special ring-shaped force sensors [37], and another study measured the right thumb compression force exerted to hold the instrument using a piezoresistive sensor [33]. In both cases, the chosen instrument was the clarinet. Although the right arm and hand in woodwind players are the most frequently injured body parts due to the weight bearing of the instrument [8], only two of the studies evaluated the finger force during performance [33,37]. This finding could lead future research to contribute to increasing the number of studies.

The research demonstrated that pressure sensors allow quantifying the force exerted, improving the understanding of musculoskeletal demands during the execution of the instrument. They are also useful as a complementary test for the diagnosis of disorders of the orofacial musculature and TMJ.

### 4.2. Surface Electromyography

SEMG represented the second most used method [32,33,34,35,36,38]. The studies mainly focused on measuring the muscle activity of the upper extremity [33,35,38] and the face [32,36].

The studies showed that SEMG could quantify the activity of a large variety of muscles under different conditions, although only one study [32] examined a group of musicians that had reported a pathology (specifically pain in the TMJ and jaw muscles). Thus, the studies considered in this systematic review sustain important methodological differences that limit the comparability of the results. For example, two of the studies analyzed the activity of the masseter muscles [32,36]: the first one [36] evaluated the activity in the right side of the face in a group of healthy clarinetists that played a musical piece and two scales, whereas the other study [32] involved a larger group of participants consisting of various woodwind and brass specialties, with pain in the TMJ and jaw muscles, playing a specific pitch, a pitch one octave higher and a repertoire of 90 min.

In accordance with the aforementioned, wind musicians may present muscular hyperactivity due to the effort exerted by the perioral structures during embouchure [13,25]. Similarly, the muscle activation patterns of the upper extremity have also received interest from researchers. In this regard, a review by Overton et al. [17] on evidence of electromyographic muscle activity in the neck, shoulder, and spinal musculature of musicians found conflicting evidence that related pain to an increase of the muscle activity in the neck and shoulder musculature (upper and lower trapezius, upper cervical extensors and sternocleidomastoid muscle). The researchers concluded that further studies were warranted to better understand the relationship between pain and muscle activity in musicians [17].

Another aspect of EMG to highlight is the possibility of biofeedback, a process in which, while the musician performs, he can graphically see the behavior of his musculature and make corrections of an incorrect technique [16], favoring the reduction of muscular tension or better performance [41]. None of the studies included in this review used EMG biofeedback.

Finally, it should be noted that there was only one study [33] that simultaneously applied the two most widely used methods according to the findings of this review. These are SEMG and pressure sensors. Therefore, it would be advisable to carry out more research that analyzes the musculoskeletal demands during musical performance through the analysis of muscle activity patterns with SEMG, combined with the quantification of force with pressure sensors, either while supporting the weight of the instrument or during the embouchure, in order to establish possible correlations.

### 4.3. Infrared Thermography

IT was the third most used method, and all the studies evaluated the thermal patterns of regions of the cranio–cervical–mandibular complex (CCMC) [25,27,30,31,39].

The included studies demonstrated that IT is useful for the assessment of CCMC regions [27] and as a complementary tool for the diagnosis of malocclusion and TMD [30,31]. However, the number of participants was very small. In addition, due to the scarce methodological information, the results cannot be compared. It should also be added that none of the studies considered applied IT to assess areas of the body other than those of the CCMC. Future research could use IT as a complementary test to diagnose other pathological conditions, such as inflammatory diseases, overload, and muscle fatigue, in other body regions, such as the upper extremity.

### 4.4. Kinematic Studies

Kinematic studies for the assessment of posture and movement [35,40] were the least frequent. One research conducted a three-dimensional analysis with UT in saxophonists [40]. Another study used two-dimensional goniometric analysis to evaluate the sitting posture in clarinetists [35], but the research did not specify which program was used.

An earlier review by Schemmann et al. [5] identified two studies that combined UT and SEMG in violinists. Additionally, Kelleher et al. [1] concluded that one of the most commonly used methods to analyze movement in string musicians (violinists, violists, cellists, and double bassists) was photogrammetry, which captures movement using cameras and reflective markers. However, according to the findings of this review, no study used photogrammetry to analyze movement in woodwind players.

### 4.5. Implications for Future Research

There is great heterogeneity in the studies included in this review. Furthermore, due to the scarce number of participants and the lack of methodological information, the results cannot be compared. Additionally, the small number of studies found for each of the biomechanical methods instills the need to increase both their quantity and their quality in future research. This may have important implications in the treatment and prevention of injuriesor MSDs related to musical practice, as well as in artistic performance.

All of this could allow us to achieve a methodological standardization that would take into account the biomechanical method used, the musical instrument played, and the pathology, if applicable. For example, the most convenient way to place the pressure sensors in the embouchure could be to establishor determine which muscles should be evaluated to discover their activation patterns based on a certain pathology or MSD.

## 5. Conclusions

Pressure sensors, SEMG, IT, 2D goniometry, and 3D UT are biomechanical methods useful to broaden the knowledge of musculoskeletal demands during a musical performance. A Piezoresistive pressure sensor is the most widely used method.

The studies included in this systematic review were very heterogeneous and few. For this reason, it is difficult to compare their results. Instead, itis necessary to increase the size and quality of research in this area for better knowledge of the musculoskeletal demands of woodwind musicians and thus be able to develop strategies for the prevention and treatment of injuries or MSDs related to musical practice.

## Figures and Tables

**Table 2:** Summary of selected studies.

Authors (Year)	Biomechanical Methods Used	Objectives	Method (Musical Activity)	Other Evaluations	Results	Conclusions	STROBE Total Score
Ackermann et al. (2014) [34]	SEMG	Investigate respiratory movements and abdominal muscle activity.	Playing five musical excerpts in four different postures (sitting flat, sitting inclined forward, sitting inclined backward and standing).	RIP	Higher chest cavity expansion standing (*p* < 0.01) and lower abdominal cavity expansion in sitting postures (*p* < 0.01). Lower activation in seated postures in comparison to standing posture (*p* < 0.01).	Significant differences in respiratory mechanics between sitting and standing postures.	12
Baadjou et al. (2017) [35]	2D Goniometry SEMG	Analyze the relationship between body posture, muscle activity, and sound quality.	Playing a 60-s musical excerpt in two different postures (habitual sitting posture and experimental sitting posture).	Not carried out.	Smaller low thoracic angle, smaller high thoracic angle, and larger pelvic tilt angle in the experimental posture (*p* < 0.001). More activity of the erector spinal and lower trapezius muscles and less activity of the left upper trapezius and right brachioradialis muscles in the experimental sitting posture.	Postural exercise therapy may change muscle activity patterns.	13
Barros et al. (2018) [25]	IT	Analyze and record the thermal patterns of the CCMC to evaluate its structures.	Before and after playing a musical piece (“Vingt Etudes”) for 10 min.	Questionnaire (musical and clinical history of the participant) and clinical examination.	Statistically significant differences (*p* < 0.05) between before and after musical in the left temporal muscle, orbicularis muscle, perioral teguments, and teeth 11 and 21. Asymmetries ≥0.3 °C in the temporal and the orbicularis muscles at rest position and after the musical performance.	IT has been proven to be an effective complementary diagnostic tool in the monitorization of the CCMC.	10
Clemente et al. (2018) [29]	Pressure sensors	Quantify the pressure applied to the central incisors during embouchure.	Playing three times three different pitches (high, medium, and low).	Clinical and radiographic examination.	Greater force was applied during lower-pitched notes, especially to tooth 11 (108 g).	Pressure sensors are acceptable for identifying the tooth where the greatest pressure is applied.	10
Clemente et al. (2018) [30]	Pressure sensors IT	Analyze the morphological and functional aspects of the CCMC with and without a mouthpiece.	Force: playing three times three pitches (high, medium, and low). IT: after playing Ode to Joy during 5 min.	Clinical examination. Cephalometric analysis.	Greater pressure on the lower lip with the English horn and in the upper lip with the oboe. Difference of 0.3 °C between the right TMJ (34.7 °C) and the left TMJ (35 °C). Difference of 0.3 °C between the right masseter (35.4 °C) and the left masseter (35.1 °C).	Pressure sensors and IT can beuseful screening tools forthe diagnosis of TMDs.	9
Clemente et al. (2018) [31]	Pressure sensors IT	Describe the steps in the diagnosis and treatment of TMDs.	Pressure: performing three times three different pitches (high, medium, and low). IT: before and after using an occlusal splint during 6 months.	Clinical examination.	Higher pressure in higher pitches (94 g in tooth 11 y 408 g in tooth 21). Thermic difference between left and right side of the masseter muscle of 0.7 °C before and 0.3 °C after the use of the splint.	Pressure sensors and IT are useful in the diagnosis and monitoring of TMDs.	10
Clemente et al. (2019) [26]	Pressure sensors	Quantify the applied forces of the perioral structures during embouchure.	Playing three times three different pitches (high, medium, and low).	Not carried out.	F-mean (upper sensor/lower sensor) in clarinet (58 g/54.1 g), oboe (23 g/17 g), saxophone (38.9 g/62.7 g), bassoon (6.3 g/10.3 g). F-mean (lower sensor) in bisel flute (73 g) and transverse flute (220 g). F-mean (upper sensor/lower sensor) in trumpet (62.7 g/89.2 g), french horn (56 g/86 g), and trombone (201 g/220 g).	Brass players apply greater force than woodwind players during embouchure.	8
Clemente et al. (2019) [27]	Pressure sensors IT	Demostrate the usefulness of pressure sensors and IT as complementary diagnostic tools during embouchure.	Force (clarinet): playing a musical piece in a high pitch. IT (tuba): N/A.	Clinical examination. Cephalometric analysis (bassoon).	Asymmetric force in the two upper central incisors (2.5 N in tooth 21). Asymmetries of 0.4 °C between the left masseter muscle (32.6 °C) and the right one (33.0 °C). Difference of 0.3 °C between the left temporal muscle (33.3 °C) and the right one (33.6 °C). Difference of 0.3 °C between the left TMJ (32.6 °C) and the right TMJ (32.9 °C).	Pressure sensors and IT can be considered as complementary diagnostic tool.	9
Clemente et al. (2019) [28]	Pressure sensors	Measure forces at the lower lip during embouchure.	Playing three times three different pitches (high, medium, and low).	Clinical examination.	F-mean (lower sensor) in clarinet (58.8 g). F-mean (lower sensor) in saxophone(94 g).	Pressure sensors allow measuring the forces at the lower lip.	8
Clemente et al. (2020) [39]	IT	Assess regions of interest of the CCMC to evaluate muscular hyperactivity.	N/A	Not carried out.	Asymmetries ≥0.3 °C in the anterior temporal muscle between wind and string instrumentalists. Statistical significant differences (*p* = 0.044) in the anterior triangle of the neck between wind and string instrumentalists.	IT can be considered as a complementary diagnostic method.	12
Franz et al. (2020) [36]	SEMG	Identify the facial muscle activity patterns involved in playing and compare them between students and professionals.	Playing a musical piece and two scales	Not carried out.	Higher activity for the masseter (*p* = 0.0007), buccinator (*p* = 0.0001) and mylohyoid (*p* = 0.000) in students and for the mentalis in professionals (*p* = 0.000).	Significantly higher facial muscle activity in students.	17
Gotouda et al. (2007) [32]	SEMG	Analyze the influence of pitch changes on the activity of jaw-closing muscles. Elucidate the effect of sustained playing on fatigue of the jaw-closing muscles.	Test 1 (N = 33): playing a tuning tone and a pitch an octave higher and under other conditions (rest, clenching, and open-mouthed). Test 2 (N = 18): before and after playing non-stop for 90 min.	Questionnaire to measure the prevalence of musculoskeletal symptoms.	Test 1: higher RMS in high pitch in brass (*p* < 0.05). Higher RMS in high pitch in woodwind. Test 2: non-significant differences between groups.	Contraction load to jaw-closing muscles when playing a wind instrument is very small. Playing for a long time does not obviously induce fatigue.	14
Hofmann y Goebl (2016) [37]	Pressure sensors	Measure finger force while playing.	Test 1: playing eight selected excerpts from the first Weber concerto under controlled different performing conditions. Test 2 (technical exercise): playing an isochronous 23-tone melody in different tempos.	Articulatory tongue-reed interactions (with strain gauge sensors). Questionnaire (self-evaluation of finger forces; discomfort).	Test 1: F-mean = 1.17 N. Test 2: F-mean = 0.64 N (0.54 N in professionals and 0.68 N in students; *p* = 0.213).	Sensor-equipped instruments help to understand fine motor actions.	16
Piatek et al. (2018) [40]	3D UT	Examine the influence of three different saxophone-carrying systems (neck-strap, shoulder-strap, and Saxholder) on the kinematics of the spine.	Playing 3-min pieces of music with and without each carrying system. Exam 1 (N = 14): alto saxophone. Exam 2 (N = 1): saxophone (alto, tenor, and baritone).	BMI	Head bows forward at a greater angle (3.35°) using a shoulderstrap than using a Saxholder (*p* = 0.02).	UT allows to investigate the influence of instrument-carrying systems on the kinematics of the spine.	12
Smyth y Mirka (2021) [33]	SEMG Pressure sensors	Determine the impact of the neck strap on thumb force while measuring the thenar, cervical, and shoulder muscle activity.	After playing a set of exercises during 3 min with and without a neckstrap.	Perceived effort survey using a scale from 0 (no effort) to 5 (severe effort).	Non-statistically significant increases in the muscle activity of any muscles of the neck, the shoulder, or the thenar muscles with the neck strap (*p* > 0.05). Significant decrease in average thumb force with the neck strap (*p* < 0.05).	The use of a neck strap significantly decreases the average force of the right thumb.	12
Young y Winges (2017) [38]	SEMG	Address the impact of the thumb-rest position on the neuromuscular control of holding the instrument.	Performing 10 held notes and 10 exercises on three different thumb-rest positions (low, traditional, and high).	Not carried out.	Significantly decreased activity of the abductor pollicis brevis and the flexor carpi ulnaris in a high thumb-rest position.	Adjustment of the thumb-rest position may alleviate discomfort in the supporting limb.	13

2D, two dimensions; 3D, three dimensions; TMJ, temporomandibular joint; CCMC, cranio-cervical-mandibular complex; SEMG, surface electromyography; F-mean, mean force; BMI, body mass index; N/A, non-available; RIP, respiratory inductive plethysmography; RMS, root mean square; IT, infrared thermography; TMDs, temporomandibular disorders; UT, ultrasound topometry.

**Table 3:** Category of biomechanical assessments and number of studies using it.

Category	Method	Number of Studies	Reference
Kinetics	Pressure sensors	8	Clemente et al. (2018) [29]; Clemente et al. (2018) [30]; Clemente et al. (2018) [31]; Clemente et al. (2019) [26]; Clemente et al. (2019) [27]; Clemente et al. (2019) [28]; Hofmann and Goebl (2016) [37]; Smyth and Mirka (2021) [33]
Kinematics	2D Goniometry	1	Baadjou et al. (2017) [35]
	3D UT	1	Piatek et al. (2018) [40]
Physiology	SEMG	6	Ackermann et al. (2014) [34]; Baadjou et al. (2017) [35]; Franz et al. (2020) [36]; Gotouda et al. (2007) [32]; Smyth and Mirka (2021) [33]; Young and Winges (2017) [38]
	IT	5	Barros et al. (2018) [25]; Clemente et al. (2018) [30]; Clemente et al. (2018) [31]; Clemente et al. (2019) [27]; Clemente et al. (2020) [39]

2D, two dimensions; 3D, three dimensions; SEMG, surface electromyography; IT, infrared thermography; UT, ultrasound topometry.

**Table 4:** Muscles that were analyzed by SEMG.

Muscles	Reference
Abdominal muscles.	Ackermann et al. (2014) [34]
Erector espinae, latissimus dorsi, low trapezius, upper trapezius, pectoralis major (clavicular head), biceps brachii (short head), and brachioradialis.	Baadjou et al. (2017) [35]
Sternocleidomastoid, masseter, mentalis, mylohyoid and right side buccinator	Franz et al. (2020) [36]
Masseter, temporal, orbicularis oris, and left side digastric (test 1); left masseter (test 2).	Gotouda et al. (2007) [32]
Trapezius, semispinalis, and sternocleidomastoid; thenar muscle group of the right thumb.	Smyth and Mirka (2021) [33]
Triceps brachii, biceps brachii, extensor carpi radialis longus, flexor carpi ulnaris, brachioradialis, extensor pollicis brevis, abductor pollicis brevis, and first dorsal interosseou muscle of the right hand.	Young and Winges (2017) [38]

## Data Availability

Data are available upon request to the first author (J.L.-P.).
